# Selenoprotein M Protects Intestinal Health in Nickel-Exposed Mice: Implications for Animal Welfare Under Heavy Metal Stress

**DOI:** 10.3390/vetsci12100955

**Published:** 2025-10-04

**Authors:** Qiaohan Liu, Kaixuan Zhang, Hongxue Yang, Xuehan Jiang, Yi Fang, Jingzeng Cai, Ziwei Zhang

**Affiliations:** 1 College of Veterinary Medicine, Northeast Agricultural University, Harbin 150030, China; liuqiaohan61@163.com (Q.L.); zhangkaixuan202202@163.com (K.Z.); 18249673863@163.com (H.Y.); xuehan415@163.com (X.J.); 19917621196@163.com (Y.F.); caijingzeng@neau.edu.cn (J.C.); 2 Key Laboratory of the Provincial Education, Department of Heilongjiang for Common Animal Disease Prevention and Treatment, Harbin 150030, China

**Keywords:** SelM, NiCl_2_, oxidative stress, autophagy, colonic inflammation, mouse

## Abstract

**Simple Summary:**

Heavy metal pollution poses a growing challenge to both human and animal health. Nickel, although required in trace amounts, becomes harmful when present in excess, particularly affecting the digestive system. In this study, we investigated how a specific selenium-containing protein, called selenoprotein M (SelM), protects the colon when animals are exposed to nickel. We used mice that lacked SelM as well as normal mice, and we also carried out cell culture experiments. Our results showed that nickel exposure caused damage in the colon by increasing harmful molecules known as reactive oxygen species, overstimulating a cellular process called autophagy, and triggering inflammation. These harmful changes were much stronger in animals without SelM. On the other hand, SelM helped maintain a healthier balance in the colon by reducing oxidative stress and controlling inflammation. These findings suggest that selenium and its related proteins may help protect the gut against heavy metal pollution. Such insights are valuable for improving animal health and welfare, and they may also be useful for developing strategies to safeguard livestock production under environmental stress.

**Abstract:**

Nickel (Ni) is a heavy metal element and environmental pollutant that significantly threatens human health. Selenoprotein M (SelM) is a selenium-containing protein with antioxidant properties. However, the role of SelM deficiency in Ni -induced colonic tissue damage in mice remains unclear. To address this, in vivo and in vitro models were established, including SelM knockout (SelM^(−/−)^) and/or nickel chloride (NiCl_2_)-treated mice. In vitro, an MCEC model was used to establish Ni exposure and SelM knockdown conditions. The results showed that NiCl_2_ induced significant inflammatory cell infiltration and lesions in the microstructure of the mouse colon. Additionally, Ni exposure was found to enhance the production of reactive oxygen species (ROS) in mice’s colonic tissue, activating oxidative stress, which in turn led to the formation of autophagosomes and the onset of inflammation. Significantly, SelM knockout exacerbated these outcomes. The oxidative stress inhibitor NAC and the autophagy inhibitor 3-MA were introduced to elucidate the underlying mechanisms further. The results showed that autophagy was reduced following NAC treatment, and inflammation was alleviated after 3-MA administration. Taken together, these findings suggest that SelM alleviated Ni -induced colonic inflammation in mice through suppression of oxidative stress-mediated excessive autophagy.

## 1. Introduction

Nickel (Ni), with an average crustal abundance of 0.008% [1], has become a pervasive environmental pollutant—contaminating soil and accumulating in industrial products worldwide. Studies indicate that heavy-metal pollution spans roughly 20 million hectares globally, with Ni being a major contributor. Its widespread industrial applications—in metallurgy, electronics manufacturing, and medical devices—further exacerbate environmental Ni dissemination [2]. Although Ni serves as a physiological trace element, its excessive accumulation exerts broad toxicity across multiple tissues, including the lungs, kidneys, liver, skin, and intestines [3]. Our prior work in mice confirmed Ni-induced injury in the heart, liver, and nervous system [4,5,6]. Notably, the gastrointestinal (GI) tract—responsible for digestion, absorption, secretion, motility, and immune defense—is also susceptible to Ni toxicity. Moreover, Ni exposure has been shown to significantly impair intestinal integrity. It can induce apoptosis in intestinal epithelial and immune cells, alter the expression of key immune molecules and enzymes, disturb the gut microbial ecosystem, and ultimately disrupt the structural and functional stability of the intestinal barrier [7,8]. Animal welfare has become a critical concern in modern livestock production, especially under increasing environmental stressors such as heavy metals. Nickel (Ni), a common pollutant in feed and water, poses significant threats to intestinal health and immune homeostasis in animals. Research indicates that Ni chloride may induce apoptosis in chicken small intestinal cells by attenuating Bcl-2, Bax, and caspase-3 expression levels and enhancing the cytochrome C, Bak, and caspase-9 [9]. Wu et al. reported that Ni particles worsen Crohn’s disease by interfering with the autophagy process in mouse intestines [10].

Autophagy is a highly conserved cellular self-repair mechanism characterized by the lysosomal degradation of long-lived proteins, misfolded aggregates, and dysfunctional organelles. This process plays an essential role in maintaining intracellular homeostasis, especially under stress conditions such as nutrient deprivation, hypoxia, and oxidative stress. As a tightly regulated catabolic pathway, autophagy contributes to cell survival by removing cytotoxic components and recycling metabolic substrates [11,12]. Among various stress stimuli, reactive oxygen species (ROS) serve as key upstream signals that initiate autophagy. ROS-induced autophagy can be mediated through multiple signaling cascades, including autophagy-related gene 4 (ATG4), the AKT/mTOR axis, MAPK/high mobility group box 1 (HMGB1), and alterations in mitochondrial electron transport chain activity [13]. Although autophagy is generally considered cytoprotective, sustained or excessive autophagic activity can paradoxically lead to autophagy-associated cell death and exacerbate tissue injury. For instance, cadmium-induced oxidative stress increases LC3-II expression and autophagic flux, leading to cytotoxicity in chicken embryonic fibroblasts [14]; lead exposure activates the PINK1/Parkin-mediated mitophagy pathway in both HEK293 cells and the renal cortex of mice, contributing to mitochondrial degradation and tissue damage [15], Likewise, Ni exposure has been shown to trigger oxidative stress-induced neuronal autophagy via the PI3K/AKT/mTOR pathway, resulting in neurotoxicity in murine models [4]. In addition to its effects on autophagy, Ni is also known to provoke inflammatory responses across multiple organ systems. Zhao et al. demonstrated that Ni sulfate induces ovarian fibrosis and inflammation by activating the transforming growth factor-β1 (TGF-β1) and nuclear factor kappa-light-chain-enhancer of activated B cells (NF-κB) signaling pathways [16]. Similarly, Kinbara et al. reported that intradermal injection of NiCl_2_ into mouse ears led to marked allergic inflammation, indicating a strong pro-inflammatory potential of Ni compounds [17]. Ni exposure has also been implicated in the disruption of intestinal epithelial barrier integrity, thereby promoting intestinal inflammation and systemic immune dysregulation [8]. Moreover, recent studies suggest a complex interplay between autophagy and inflammation. While basal autophagy helps suppress inflammatory responses by limiting inflammasome activation and removing pro-inflammatory substrates, uncontrolled autophagy may have the opposite effect; excessive autophagy under persistent stress conditions has been shown to stimulate inflammatory responses by promoting cytokine release and disrupting immune regulation [18]. Martinet et al. revealed that excessive autophagy contributes to endothelial cell apoptosis and plaque instability in atherosclerosis, thereby aggravating inflammation [19]. Alexandra et al. further confirmed that persistent autophagy can lead to pulmonary epithelial damage and inflammation during lung injury [20]. These observations collectively underscore the dual role of autophagy: it acts as a protective mechanism under controlled activation but may contribute to pathological outcomes when dysregulated. In the context of Ni-induced toxicity, both autophagy and inflammation are prominent pathological features, yet their mechanistic relationship in intestinal tissue remains largely unexplored. Although selenium, an essential micronutrient, has demonstrated protective effects against heavy metal-induced oxidative and apoptotic damage through its incorporation into selenoproteins and redox enzymes [21,22,23], whether it modulates autophagy in Ni-induced intestinal inflammation has not been fully elucidated.

Selenoprotein M (SelM), situated within the endoplasmic reticulum, serves as a key mediator of selenium’s biological functions. As a SelM/SEP15 family constituent, it is implicated in a spectrum of biological processes, exhibiting antioxidant, neuroprotective, and intracellular calcium regulatory functions. It is widely expressed in the brain, kidneys, liver, lungs, heart, and intestines [24], with exceptionally high expression in brain tissue [25]. Studies have indicated that SelM is involved in oxidative stress, immune responses, endoplasmic reticulum stress, and regulation of calcium ion homeostasis [26,27]. SelM contains a redox-active CXXU group, a characteristic of enzymes involved in regulating redox processes. SelM has been implicated in tissue injury induced by various toxic agents [28]. Furthermore, SelM can improve metabolic disorders by regulating endoplasmic reticulum and inflammatory stress responses [24]. Our previous studies revealed that Selenoprotein M (SelM) is downregulated in the liver of high-fat diet-fed mice, and its knockdown exacerbates hepatic steatosis, oxidative stress, and fibrosis [22]. Using a SelM-knockout mouse model generated via CRISPR/Cas9, we further confirmed its antioxidant function, showing that SelM deficiency intensifies endoplasmic reticulum (ER) stress and apoptosis in the heart [6], and promotes splenic necrosis and tissue damage under Ni exposure through oxidative mechanisms [29]. These findings highlight SelM’s protective role in multiple organs under pathological stress. Despite this, SelM’s role in gastrointestinal defense—particularly under heavy metal exposure—remains uncharacterized. Some selenoproteins, including SelM, are expressed in intestinal epithelial cells and contribute to redox regulation, barrier integrity, and immune balance. Prior studies have shown that selenium supplementation alleviates colitis and restores tight junction proteins like ZO-1, supporting a broader protective role for selenoproteins in the gut.

Based on these findings, we hypothesized that SelM may also protect the intestine from Ni-induced damage. To test this, we established a SelM-knockout model combined with chronic Ni exposure. In vivo experiments assessed intestinal autophagy and inflammation, while in vitro assays explored the underlying mechanisms, including the use of oxidative stress and autophagy inhibitors. Our results show that SelM knockout aggravates Ni-induced oxidative stress, enhances autophagy, and worsens inflammation in murine colonic tissue. This study identifies a novel role for SelM in protecting the intestine from heavy metal toxicity and offers insight into potential molecular targets for intervention in Ni poisoning.

## 2. Materials and Methods

All animal experimental protocols in this investigation adhered to the Northeast Agricultural University’s Institutional Animal Care and Use Committee guidelines (NEAUEC202403101).

### 2.1. Treatment of Experimental Animals

Eighty wild-type (WT) and SelM knockout (SelM^(−/−)^) C57BL/6J mice, including both males and females (weighing 25–30 g), were used. The animals were randomly allocated into four groups: wild-type control (WT), wild-type Ni (Ni), knockout control (SelM^(−/−)^), and knockout Ni (SelM^(−/−)^ + Ni), with 20 mice per group. Among the 20 animals in each group, six biological replicates (n = 6) were randomly selected and analyzed individually for molecular and histological assays. The remaining animals were reserved for replication of key findings and for additional assays such as Western blotting, qRT-PCR, and biochemical analyses to ensure data reliability. The control group received 0.9% saline, whereas the Ni group received a 10 mg/kg dose of Ni chloride, administered by oral gavage at a volume of 0.1 mL per 10 g body weight. The NiCl_2_ concentration was chosen based on a previous report [30]. After 21 days of treatment, the mice were euthanized, and colonic tissues were collected for the analysis of autophagy and inflammatory markers.

### 2.2. In Vitro Cell Culture

Mouse colon epithelial cells (MCECs) were cultured in DMEM (catalog no. P150210) supplemented with 20% fetal bovine serum (FBS) at 37 °C in a humidified incubator with 5% CO_2_. When the cells reached approximately 70% confluence, they were transfected with 50 nM SelM-targeting siRNA (5′-GCCGAAATTACCAGGAACTAG-3′) or negative control siRNA (si-NC), purchased from Guangzhou RiboBio Co., Ltd. (Guangzhou, China), using Lipofectamine 3000 (Invitrogen) according to the manufacturer’s instructions. After 12 h of transfection, the medium was replaced, and the cells were washed twice with sterile PBS, then incubated in fresh complete medium containing 20% FBS for an additional 24 h. For subsequent experiments, cells were divided into different treatment groups. The Ni and si-SelM + Ni groups were treated with 5 μM nickel chloride (NiCl_2_) for 12 h. In the si-SelM + Ni + NAC group, cells were pretreated with 2.5 mM N-acetylcysteine (NAC) for 2 h, followed by NiCl_2_ treatment (5 μM) for 12 h. Similarly, in the si-SelM + Ni + 3-MA group, cells were pretreated with 5 mM 3-methyladenine (3-MA) for 2 h prior to NiCl_2_ exposure. The concentrations of NiCl_2_, NAC, and 3-MA used in these experiments were selected based on published literature and validated through preliminary CCK-8 assays to ensure appropriate cell viability [31]. The treatment groups for in vitro cell culture experiments are summarized in Supplementary Table S1.

### 2.3. Histological Observation of Colonic Tissue

Fresh colonic tissues were fixed in 10% neutral-buffered formaldehyde for 24–48 h. Dehydration was carried out in a tissue processor using a graded ethanol series as follows: 75% ethanol (4 h), 85% ethanol (2 h), 90% ethanol (2 h), 95% ethanol (1 h), absolute ethanol I (30 min), absolute ethanol II (30 min). Clearing was then performed sequentially in ethanol/xylene (1:1, 5–10 min), xylene I (5–10 min), and xylene II (5–10 min), followed by three changes in molten paraffin at 65 °C (1 h each).

After dehydration, tissues were embedded in paraffin. Molten paraffin was poured into embedding molds, and tissues were carefully positioned according to orientation requirements. Labeled cassettes were then placed on a –20 °C cold plate until the paraffin solidified. Once solidified, paraffin blocks were trimmed and sectioned at 4 μm thickness using a rotary microtome. The tissue ribbons were floated on a 40 °C water bath, transferred to glass slides, and dried in a 60 °C oven. For histological examination, 5 μm paraffin sections were stained with hematoxylin and eosin (H&E), cleared, and observed under a light microscope.

### 2.4. Antioxidant Enzyme Activity and Content Assays

Oxidative stress was assessed by measuring several biomarkers, including glutathione (GSH), glutathione peroxidase (GSH-Px), total antioxidant capacity (T-AOC), total superoxide dismutase (T-SOD), malondialdehyde (MDA), inducible nitric oxide synthase (iNOS), nitric oxide (NO), and catalase (CAT). Diamine oxidase (DAO) was used as an indicator of colonic mucosal barrier integrity. Commercial assay kits for GSH, GSH-Px, T-AOC, T-SOD, MDA, NO, iNOS, DAO, and CAT were obtained from Nanjing Jiancheng Bioengineering Institute (Nanjing, China).

All assays were performed in accordance with the manufacturer’s instructions at the Science and Technology Experimental Center of Shanghai University of Traditional Chinese Medicine. Colonic tissues were homogenized using an electric homogenizer, and the supernatants were collected for biochemical analysis.

### 2.5. MDC and ROS Staining of MCEC

Autophagy was assessed using a Cell Autophagy Detection Kit (Beijing Solarbio Science & Technology Co., Beijing, China), following the manufacturer’s instructions. Monodansylcadaverine (MDC), the fluorescent probe used in this assay, is a lysosomotropic dye that preferentially accumulates in autophagic vacuoles due to their acidic and lipid-rich properties. Although not exclusively specific, it is widely used as a convenient marker for autophagic vesicle detection. Cells were observed under a fluorescence microscope (Olympus IX53, Tokyo, Japan) equipped with appropriate filters (excitation 380 nm, emission 530 nm) to evaluate autophagic vesicle formation. Intracellular reactive oxygen species (ROS) levels were measured using a ROS Assay Kit (Nantong Bei’ao Epoch Biotechnology Research Institute, Nantong, China). Cells were incubated with DCFH-DA at 37 °C for 30 min, and fluorescence signals were detected using the same microscope (excitation 488 nm, emission 525 nm). Quantification of ROS-associated fluorescence intensity was performed using ImageJ software (version 1.53, National Institutes of Health, Bethesda, MD, USA).

### 2.6. Determination of Protein Content by Western Blot

Western blotting was performed following previously described procedures. In brief, mouse colonic tissues and treated MCEC were lysed in ice-cold RIPA buffer supplemented with PMSF protease inhibitor. After centrifugation, the supernatants were collected, and protein concentrations were measured using a BCA assay kit. Equal amounts of protein from each sample were loaded onto 6–15% SDS-PAGE gels, separated by electrophoresis, and transferred onto NC membranes. The membranes were blocked with 5% non-fat milk in TBST at 37 °C for 2 h, then incubated overnight at 4 °C with primary antibodies against ATG7, Beclin1, LC3-II, P62, TNF-α, IL-1β, NF-κB, IL-10, and β-actin. After three washes with TBST, the membranes were incubated with HRP-conjugated secondary antibodies at room temperature for 2 h. Protein bands were visualized using an ECL detection kit and imaged with the Azure Imaging Biosystem C300.

### 2.7. Determination of mRNA Content by qRT-PCR

Total RNA was isolated from murine colon tissues and treated MCEC utilizing Trizol reagent, with RNA concentration and purity assessed via spectrophotometry at 260/280 nm. Synthesis of first-strand cDNA for qRT-PCR was conducted using a proprietary kit. This study identified autophagy-associated genes (ATG5, ATG7, Beclin1, LC3, ATG1, mTOR, and PI3K) and genes linked to inflammation (NF-κB, iNOS, COX-2, TNF-α, IL-1β, IL-2, IL-6, IL-18, IL-17, IFN-γ, and IL-10). Primers were devised utilizing online primer design tools and are delineated in Table 1. Following the preparation of the premix system (comprising diluted cDNA (1 μL), 2× SYBR Green PCR Master Mix (5 μL), PCR-grade water (3.4 μL), and primers (0.3 μL, 10 μM each)), mRNA expression levels were quantified employing the BioRT Real-Time RT-PCR Kit on a LineGene 9600 Plus apparatus. Thermal cycling conditions were as follows: initial denaturation at 95 °C for 3 min; 40 cycles of 95 °C for 10 s and 60 °C for 30 s; followed by a melting curve analysis (65–95 °C, increments of 0.5 °C) to verify amplification specificity. β-actin served as the reference gene, with data normalization executed based on the 2^−ΔΔCt^ method.

### 2.8. Statistical Analysis

For experimental data accuracy, three independent trials were executed (n = 6). Data were processed utilizing GraphPad Prism software (version 8.0). The data were analyzed using a one-way analysis of variance (ANOVA) and Tukey’s multiple comparison test, and the measurements were considered significantly different when *p* < 0.05. Different letters were used to represent the significant differences. Quantitative data are presented as mean ± standard deviation.

## 3. Results

### 3.1. SelM Knockout Exacerbates Ni-Induced Colonic Tissue Pathology

**Figure 1:**
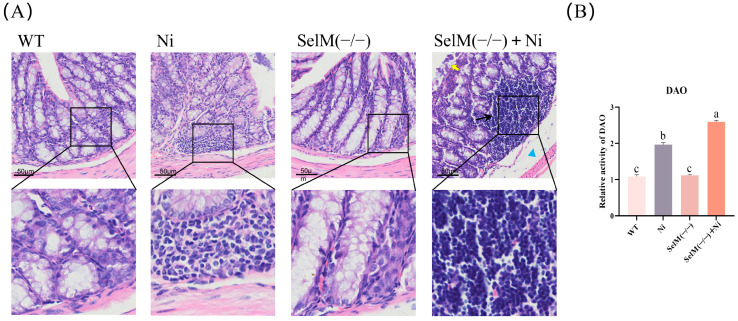
SelM knockout exacerbates Ni-induced colonic tissue pathology. (**A**) WT group and SelM^(−/−)^ group with normal structure and no apparent lesions. Ni group with mild inflammatory cell infiltration. SelM^(−/−)^ + Ni group with significant damage. Yellow arrows indicate colonic epithelial detachment. Black arrows indicate inflammatory cell infiltration. Blue squares indicate enlargement of the space between muscle layer and submucosa (n = 3). (**B**) Detection of DAO activity in colonic tissues (n = 3). Distinct letters denote significant differences (*p* < 0.05), whereas identical letters signify no significant difference (*p* > 0.05). Results are expressed as mean ± standard deviation (SD).

To assess the impacts of Ni on colonic tissues in wild-type (WT) and SelM^(−/−)^ mice, histopathological alterations were evaluated via H&E staining, observed microscopically (Figure 1). Findings indicated normal colonic tissue architecture in the WT group and SelM^(−/−)^ group, characterized by preserved epithelial integrity, absence of inflammatory cell infiltration, and lack of overt damage. The colonic tissue in the Ni group exhibited slight damage, characterized by no obvious damage to the muscular layer and submucosa, and mild infiltration of inflammatory cells. Colonic tissue in the SelM^(−/−)^ + Ni group exhibited significant damage, characterized by thinning of the muscular layer, enlargement of the gap between the muscular layer and submucosa, infiltration of inflammatory cells, detachment of the intestinal epithelium, and degeneration of epithelial cells (Figure 1A).

We explored the impairment of mucosal barrier function in mouse colonic tissues by measuring the DAO. Relative to the WT cohort, the Ni group exhibited a marked elevation in enzyme activity (*p* < 0.05). Moreover, enzyme activity in the SelM^(−/−)^ + Ni group was notably more significant compared to the Ni group (*p* < 0.05). Additionally, enzyme activity in the SelM^(−/−)^ group paralleled that of the WT group (Figure 1B). The findings imply that Ni exposure precipitates pathological alterations in murine colonic tissues, with SelM depletion intensifying these effects.

### 3.2. SelM Knockout Exacerbates Ni-Induced Oxidative Stress Parameters in Colonic Tissue and MCEC

**Figure 2:**
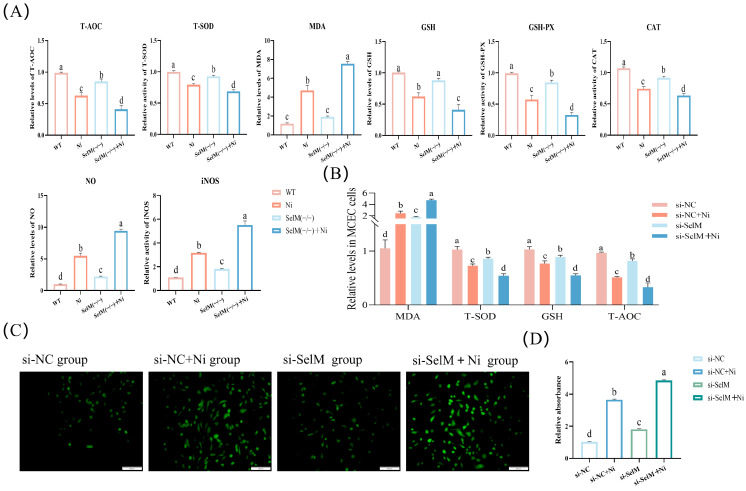
SelM knockout exacerbates Ni-induced oxidative stress parameters in colonic tissue and MCEC. (**A**) Assessment of murine colonic tissues’ levels of T-AOC, T-SOD, GSH, CAT, NO, iNOS, MDA, and GSH-Px levels (n = 6). (**B**) Quantification of MDA, T-SOD, T-AOC, and GSH levels in MCEC (n = 6). (**C**) Evaluation of ROS concentrations in MCEC (n = 6). (**D**) Quantitative assessment of ROS fluorescence intensity (n = 6). Distinct letters denote significant differences (*p* < 0.05), whereas identical letters signify no significant difference (*p* > 0.05). Results are expressed as mean ± standard deviation (SD).

We assessed the production of ROS in MCEC following Ni treatment and SelM knockout using DCFH-DA staining (Figure 2C). Findings revealed a significant elevation in ROS production in the si-NC + Ni group relative to the si-NC group (*p* < 0.05), suggesting that Ni exposure prompts oxidative stress in MCEC. Relative to the si-NC group, there was a notable rise in ROS levels within the si-SelM group (*p* < 0.05). Furthermore, ROS levels in the si-SelM + Ni group were significantly higher compared to the si-NC + Ni group (*p* < 0.05), implying that SelM depletion intensifies Ni -induced oxidative stress.

To assess the antioxidant capacity of colonic tissues and MCEC, we quantified T-AOC, T-SOD, GSH, CAT, NO, iNOS, MDA, and GSH-Px levels. In colonic tissues, the Ni group demonstrated a significant reduction in T-AOC, T-SOD, GSH, CAT, and GSH-Px levels compared to the WT group (*p* < 0.05), concurrently, NO, iNOS and MDA levels significantly escalated (*p* < 0.05). Relative to the Ni group, the SelM^(−/−)^ group exhibited markedly decreased T-AOC, T-SOD, GSH, CAT, and GSH-Px levels (*p* < 0.05), accompanied by a significant elevation in NO and iNOS levels (*p* < 0.05). Furthermore, the SelM^(−/−)^ + Ni group displayed a significant diminution in T-AOC, T-SOD, GSH, CAT, and GSH-Px levels compared to the Ni group (*p* < 0.05), with a concurrent significant increase in NO, iNOS, and MDA levels (*p* < 0.05). These findings paralleled those observed in MCEC (Figure 2B). In summary, the data suggest that Ni exposure prompts oxidative stress in mouse colonic tissues, and SelM depletion intensifies this stress.

### 3.3. SelM Knockout Exacerbates Ni-Induced Autophagy Parameters in Colonic Tissue and MCEC

**Figure 3:**
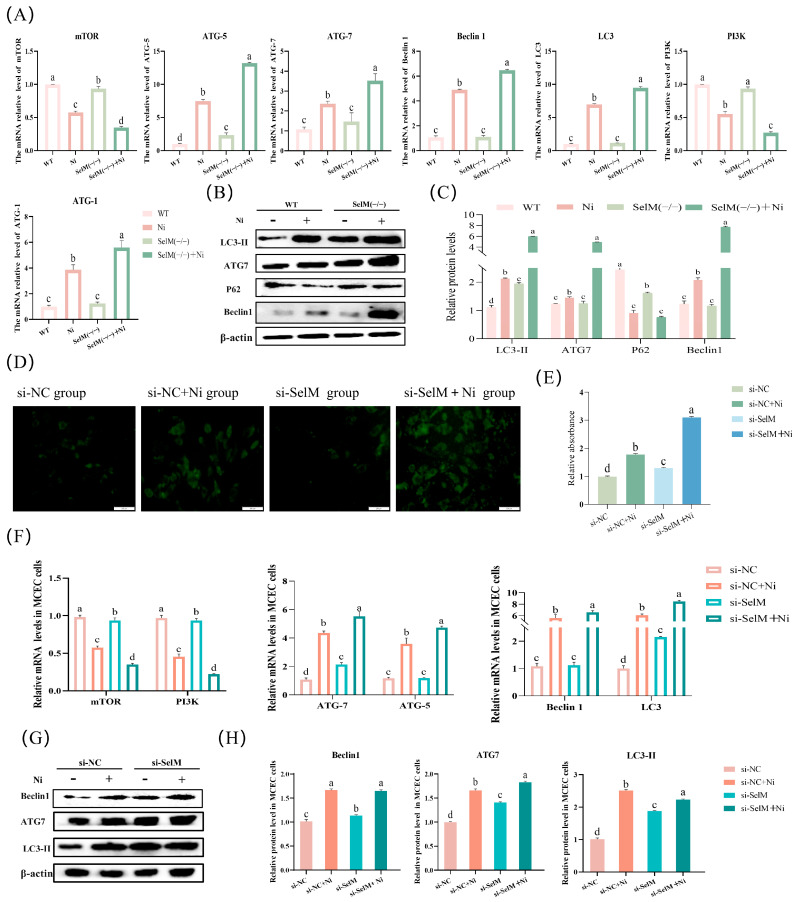
SelM knockout exacerbates Ni-induced autophagy parameters in colonic tissue and MCEC. (**A**) Assessment of mRNA expression for autophagy markers in murine colonic tissues (n = 6). (**B**,**C**) Evaluation of protein expression for autophagy markers in murine colonic tissues (n = 6). (**D**) Detection of autophagosomes in MCEC using MDC staining (n = 6). (**E**) Quantitative analysis of MDC fluorescence intensity (n = 6). (**F**) Quantification of mRNA expression for autophagy markers in MCEC (n = 6). (**G**,**H**) Quantification of protein expression for autophagy markers in MCEC (n = 6). Distinct letters denote significant differences (*p* < 0.05), whereas identical letters signify no significant difference (*p* > 0.05). Results are expressed as mean ± standard deviation (SD).

MDC, a fluorescent and acidic dye, served as a specific marker for autophagosome identification in MCEC (Figure 3D). The data revealed a significant increase in MDC-stained cells within the si-NC + Ni group compared to the si-NC group (*p* < 0.05), implying Ni exposure as an inducer of autophagy in MCEC. Additionally, the si-SelM group exhibited a significant uptick in MDC-stained cells relative to the si-NC group (*p* < 0.05). Furthermore, there was a significant elevation in MDC-stained cells in the si-SelM + Ni group compared to the si-NC + Ni group (*p* < 0.05), indicating an exacerbation of Ni-induced autophagy due to SelM depletion (Figure 3E).

This experiment measured mRNA and protein expression levels of autophagy-related genes in mouse colonic tissues and MCEC post-Ni treatment and SelM depletion. Results demonstrated that the Ni group had markedly higher mRNA expression of ATG5, ATG7, Beclin1, LC3, and ATG1 compared to the WT group (*p* < 0.05), while expressions of mTOR and PI3K were significantly lower (*p* < 0.05). Relative to the Ni group, the SelM^(−/−)^ + Ni group displayed a significant surge in mRNA expression of ATG5, ATG7, Beclin1, LC3, and ATG1 (*p* < 0.05), accompanied by a notable reduction in mTOR and PI3K mRNA expression (*p* < 0.05). These findings paralleled observations in MCEC (Figure 3F). Protein expression patterns largely mirrored mRNA expression data (Figure 3B,C). In comparison to the WT group, the Ni group presented a significant upregulation in protein expression of ATG7, Beclin1, and LC3-II (*p* < 0.05), with a corresponding significant downregulation of P62 protein expression (*p* < 0.05). The SelM^(−/−)^ + Ni group, when compared to the Ni group, demonstrated a significant augmentation in protein expression of ATG7, Beclin1, and LC3-II (*p* < 0.05), with a notable decrease in P62 protein expression also observed (*p* < 0.05). These results were consistent with those obtained in MCEC (Figure 3G,H). In conclusion, these data indicate that Ni exposure induces autophagy in mouse colonic tissues, and SelM depletion exacerbates the extent of autophagy.

### 3.4. SelM Knockout Exacerbates Ni-Induced Inflammatory Response Parameters in Colonic Tissue and MCEC

**Figure 4:**
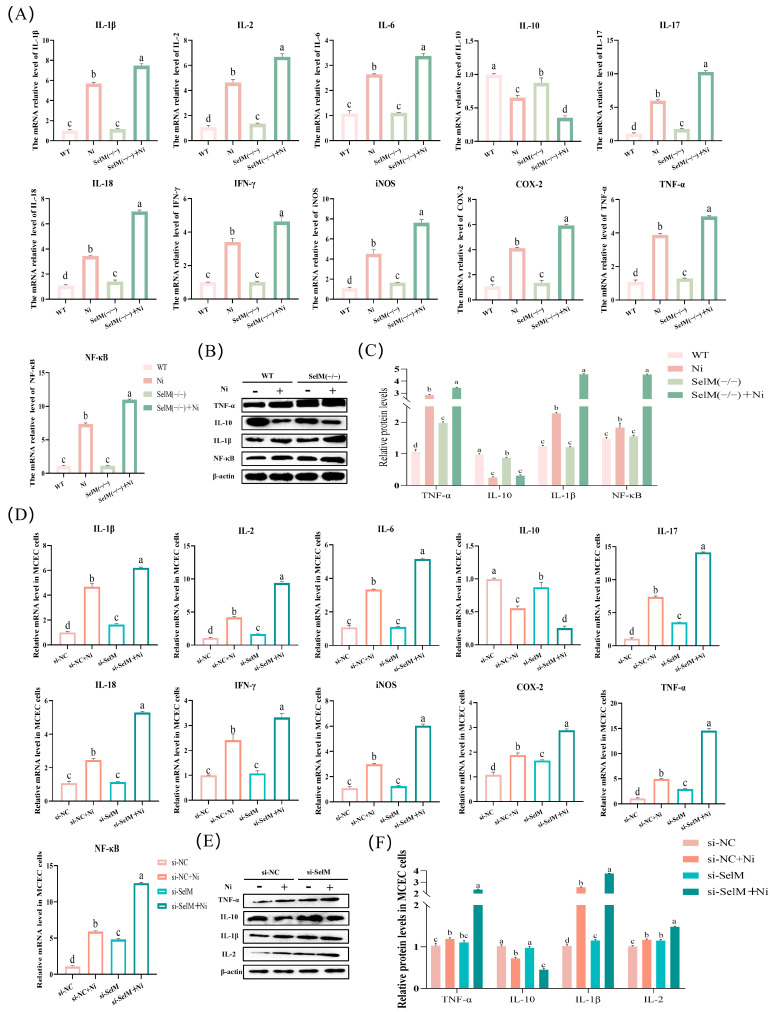
SelM knockout exacerbates Ni-induced inflammatory response parameters in colonic tissue and MCEC. (**A**) mRNA expression of inflammatory markers in murine colonic tissues was assessed (n = 6). (**B**,**C**) Protein expression of inflammatory markers in murine colonic tissues was quantified (n = 6). (**D**) mRNA expression of inflammatory markers in MCEC was evaluated (n = 6). (**E**,**F**) Protein expression of inflammatory markers in MCEC was determined (n = 6). Distinct letters denote significant differences (*p* < 0.05), whereas identical letters signify no significant difference (*p* > 0.05). Results are reported as mean ± standard deviation (SD).

In this experiment, the impact of SelM depletion on inflammatory response parameters was investigated in mouse colonic tissues and MCEC exposed to Ni. Following Ni treatment and SelM depletion, we analyzed the mRNA and protein expression of genes linked to inflammatory response markers. Results indicated a marked elevation (*p* < 0.05) in mRNA levels of NF-κB, iNOS, COX-2, TNF-α, IL-1β, IL-2, IL-6, IL-18, IFN-γ, and IL-17 in the Ni group’s colonic tissues relative to the WT group (Figure 4A). In contrast, mRNA levels of IL-10 was significantly diminished (*p* < 0.05). Relative to the Ni group, the SelM^(−/−)^ + Ni group showed an additional significant rise in mRNA expression of NF-κB, iNOS, COX-2, TNF-α, IL-1β, IL-2, IL-6, IL-18, IFN-γ, and IL-17 (*p* < 0.05), accompanied by a notable decrease in IL-10 mRNA level (*p* < 0.05). Parallel findings were noted in MCEC (Figure 4D). Protein expression outcomes generally agreed (Figure 4B,C). The Ni group’s colonic tissues displayed a significant upsurge (*p* < 0.05) in TNF-α, IL-1β, and NF-κB protein expression relative to the WT group. Conversely, the SelM^(−/−)^ + Ni group demonstrated a significant diminution (*p* < 0.05) in IL-10 protein expression compared to the Ni group. These observations were congruent with the data from MCEC (Figure 4E,F). Collectively, the data imply that Ni exposure prompts autophagy in mouse colonic tissues, and SelM depletion intensifies inflammatory response severity.

### 3.5. Effects of NAC and 3-MA on Ni-Exposed MCEC with SelM Knockdown

**Figure 5:**
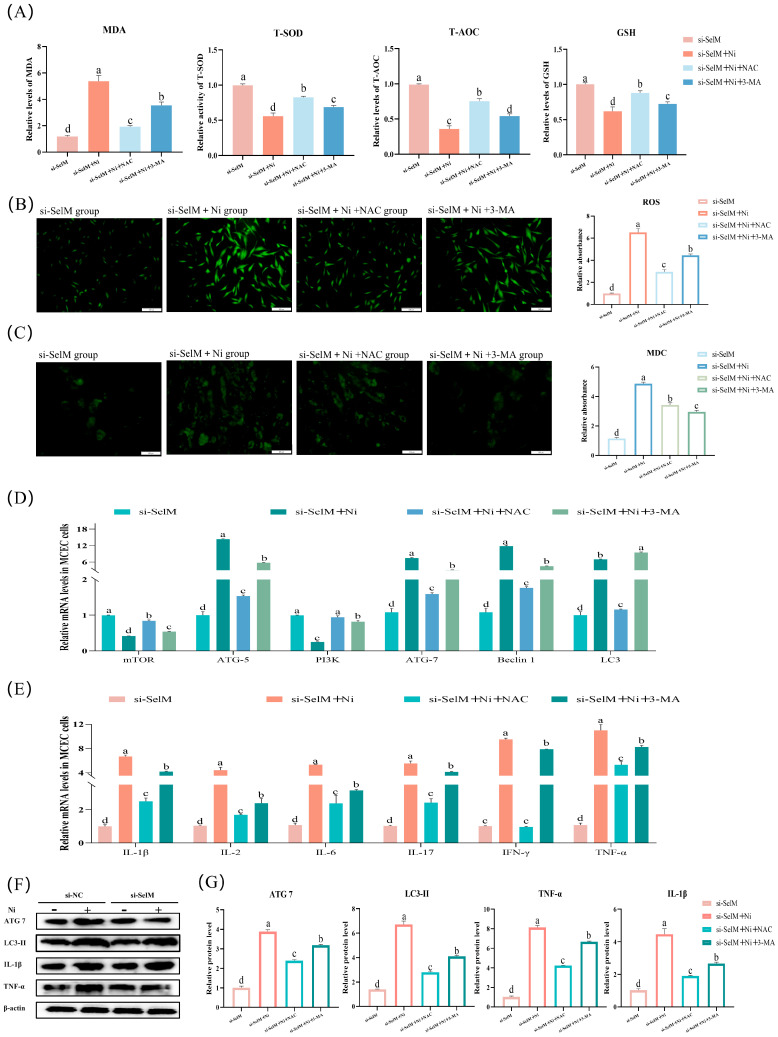
Effects of NAC and 3-MA on Ni-Exposed MCEC with SelM Knockdown. (**A**) Measurement of MDA, T-SOD, T-AOC, and GSH content in MCEC (n = 6). (**B**) Detection of ROS levels in MCEC. Quantitative analysis of ROS fluorescence intensity (n = 6). (**C**) Detection of autophagosomes in MCEC using MDC staining. Quantitative analysis of MDC fluorescence intensity (n = 6). (**D**) mRNA levels of autophagy markers in MCEC (n = 6). (**E**) mRNA levels of inflammatory markers in MCEC (n = 6). (**F**,**G**) Protein expression of autophagy and inflammatory markers in MCEC (n = 6). Distinct letters denote significant differences (*p* < 0.05), whereas identical letters signify no significant difference (*p* > 0.05). Results are reported as mean ± standard deviation (SD).

ROS and MDC staining were performed on MCEC to assess oxidative stress levels and autophagy, respectively (Figure 5B,C). Observations indicated a marked decrease in ROS production (*p* < 0.05, Figure 5B) in the si-SelM + Ni + NAC group relative to the si-SelM + Ni group. The si-SelM + Ni + 3-MA group demonstrated a notable decline (*p* < 0.05, Figure 5C) in MDC-stained cells, signifying diminished autophagy. Furthermore, we evaluated the antioxidative potential of MCEC by quantifying T-AOC, T-SOD, MDA, and GSH levels. Results (Figure 5A) revealed a significant elevation (*p* < 0.05) in T-AOC, T-SOD, and GSH levels, alongside a significant reduction (*p* < 0.05) in MDA levels in the si-SelM + Ni + NAC and si-SelM + Ni + 3-MA groups relative to the si-SelM + Ni group.

The study further investigated mRNA and protein expression of autophagy and inflammation-related genes in MCEC. mRNA expression data (Figure 5D,E) indicated a significant diminution (*p* < 0.05) in ATG5, ATG7, Beclin1, and LC3 levels in the si-SelM + Ni + NAC group, while mTOR and PI3K levels significantly rose (*p* < 0.05). Relative to the si-SelM + Ni group, the si-SelM + Ni + 3-MA group displayed a significant reduction (*p* < 0.05) in mRNA levels of TNF-α, IL-1β, IL-2, IL-6, IFN-γ, and IL-17. Protein expression findings (Figure 5F,G) demonstrated a significant decrease (*p* < 0.05) in the protein levels of ATG7, LC3-II, TNF-α, and IL-1β in the si-SelM + Ni + NAC and si-SelM + Ni + 3-MA groups relative to the si-SelM + Ni group. In conclusion, the data imply that mitigating oxidative stress in MCEC following SelM depletion and Ni exposure may attenuate the autophagic process. Moreover, dampening autophagy may partially curtail the inflammatory response.

## 4. Discussion

Ni is an essential trace metal required for various biological processes in animals; however, when accumulated in excessive quantities, it becomes toxic and damages multiple organ systems. Studies have shown that Ni exposure can lead to carcinogenesis, immunotoxicity, hepatotoxicity, and cardiovascular disorders [32]. In aquatic models, Zheng et al. observed that Ni exposure led to reduced body weight and impaired locomotor activity in fish, attributing the findings to increased oxidative stress and hepatocyte apoptosis through the JNK signaling pathway [33]. Similarly, Zhang’s study demonstrated that Ni sulfate could induce autophagy in human thyroid follicular epithelial cells [34]. Given that the colon plays a critical role in nutrient absorption and immune regulation, its structural and functional integrity is crucial for maintaining systemic homeostasis. The gastrointestinal tract, particularly the colon, is among the first tissues exposed to ingested heavy metals and thus highly susceptible to Ni-induced cytotoxicity. The selected dose of 10 mg/kg NiCl_2_ was based on previous toxicological studies [30] and is commonly used in rodent and poultry models to induce measurable intestinal injury and immune alterations [35]. Although this level is higher than typical environmental or dietary nickel exposure reported in livestock and humans [36], it provides a reliable model of acute heavy metal stress and yields mechanistic insights relevant to feed-borne nickel contamination [37]. Our study aimed to evaluate the role of the SelM in mitigating Ni-induced colonic injury. We observed that Ni exposure elevated oxidative stress, activated autophagy, and triggered inflammatory responses, all of which were aggravated upon SelM knockout, indicating its essential protective role in colonic homeostasis.

Oxidative stress is a pivotal mechanism in heavy metal-induced toxicity. The excessive accumulation of ROS can lead to severe cellular dysfunction, affecting mitochondrial respiration, redox-sensitive signaling pathways, and inducing DNA, lipid, and protein damage. In our study, Ni exposure markedly increased ROS levels in MCEC and raised MDA, NO, and iNOS activity in colonic tissues, while concurrently reducing antioxidant defenses such as T-SOD, T-AOC, CAT, GSH-Px, and GSH. These findings echo prior studies, such as Yang et al., who reported copper exposure elevated oxidative stress biomarkers including ROS, MDA, and NO in fish [38]. Similarly, Martinez-Haro et al. found that lead exposure enhanced ROS generation, further confirming heavy metals’ capacity to disrupt redox equilibrium [19]. The role of selenium in combating oxidative stress has been extensively documented. Selenium, primarily through its incorporation into selenoproteins, acts as a vital antioxidant buffer. For example, TRXR1 is involved in direct ROS detoxification and maintenance of cellular redox homeostasis [39], while selenium-binding protein S (SBP1) deficiency has been linked to mitochondrial dysfunction, loss of membrane potential, and enhanced ROS accumulation [40]. MsrB1, another selenoprotein, plays a role in repairing oxidized methionine residues, further reducing intracellular oxidative stress [41]. In our model, SelM deficiency significantly worsened oxidative parameters, suggesting that SelM exerts its protective function by modulating antioxidant capacity. Additionally, selenium’s antioxidant potential was shown in other models: Bao et al. demonstrated selenium could alleviate cadmium-induced nephrotoxicity via the PI3K/AKT/Bcl-2 pathway [42]. These data align with our observations and highlight the necessity of SelM in colon redox regulation under Ni stress.

In addition to oxidative stress, autophagy is a vital cellular mechanism activated during heavy metal toxicity. It plays a dual role by eliminating damaged organelles and misfolded proteins to sustain homeostasis, but dysregulated or excessive autophagy can promote cell death and exacerbate tissue injury. In the present study, excessive autophagy was characterized by a continuous increase in LC3-II expression coupled with persistent degradation of P62, along with concomitant inflammatory responses and histological damage. These indicators collectively reflect a shift from protective autophagy to a pathological state. Our findings indicated that Ni exposure upregulated the transcription of autophagy-related genes such as ATG5, ATG7, Beclin1, and LC3, while simultaneously reducing mTOR and PI3K expression. Protein-level validation supported these results, showing enhanced LC3-II accumulation and P62 degradation. In vitro, MCEC displayed increased autophagosome formation following Ni treatment. SelM knockout mice exhibited even higher autophagy marker expression, suggesting a suppressive role of SelM in excessive autophagic activation. Comparable results were observed by Guo et al., who found that copper ions induced autophagy in GC-1 cells and testicular tissues via an AMPK/mTOR-dependent pathway [43]. Qiao et al. demonstrated that Ni exposure impaired PI3K/Akt/mTOR signaling and promoted autophagy in murine cerebral tissues [4]. These pathways are similarly implicated in our study and collectively point toward oxidative stress as an upstream regulator of autophagy. This observation is consistent with the findings of Liang et al., who showed that cadmium exposure triggered autophagy in L-02 hepatocytes via the ROS-Akt-mTOR pathway, resulting in the accumulation of autophagic vesicles, elevated LC3-II levels, and degradation of SQSTM1/P62 [44]. This notion is supported by the regulatory actions of other selenoproteins such as SBP1, which influence mitochondrial integrity and ER stress, ultimately affecting autophagy flux [40]. Hence, SelM may inhibit pathological autophagy by preserving redox balance and mitochondrial function. These findings position SelM as an autophagy-modulating factor under Ni-induced stress.

Ni also exerts strong immunomodulatory effects, particularly within the colonic microenvironment where immune cells interact closely with the epithelial barrier. Disruption of epithelial integrity and immune balance often leads to inflammatory bowel conditions. We found that Ni exposure increased expression of pro-inflammatory cytokines and mediators including TNF-α, IL-1β, NF-κB, COX-2, IFN-γ, and iNOS, while reducing levels of the anti-inflammatory cytokines IL-10. These alterations were aggravated in SelM-deficient mice. Histological analysis revealed massive inflammatory cell infiltration and mucosal erosion, confirming colonic inflammation. Prior evidence supports these findings: cadmium exposure was shown to activate the TNF-α/NF-κB axis and upregulate inflammatory cytokines in porcine intestine [45], and ingestion of Ni particles worsened Crohn’s disease symptoms via interference with autophagy regulation in the colon [10]. Selenium’s anti-inflammatory properties are well-established. For instance, Zhang et al. found selenium mitigated lead-induced apoptosis in chicken spleen via modulation of NF-κB and activation of heat shock proteins [46], while other studies recognized selenium as a potential antidote against mercury toxicity [47]. These findings highlight the broader significance of selenium and selenoproteins in immunoregulation. Our data indicate that SelM contributes to the suppression of Ni-induced colonic inflammation by balancing immune responses and preventing epithelial injury.

To further explore the mechanistic pathways, we employed pharmacological interventions targeting oxidative stress and autophagy. Pretreatment with NAC, a well-known ROS scavenger, significantly reduced Ni-induced oxidative stress markers and autophagosome formation in MCEC. Similarly, autophagy inhibition via 3-MA decreased the expression of autophagy-related proteins and mitigated inflammatory cytokine production in both in vivo and in vitro settings. Importantly, these inhibitors were less effective in SelM knockout mice, underscoring the central role of SelM in modulating the oxidative stress–autophagy–inflammation axis. Previous reports support our findings: Murai et al. noted that autophagy promotes IL-18 secretion in respiratory epithelial cells [48], while Liu et al. demonstrated that 3-MA reduced inflammation in a model of chemically induced colitis [49]. Our work extends these observations by demonstrating that autophagy acts as a bridge linking oxidative stress and inflammatory responses in Ni-induced colonic toxicity. SelM’s absence intensified each link in this chain, leading to more severe pathological outcomes.

Comparisons with other heavy metals indicate that Ni toxicity shares common mechanisms with cadmium, copper, and lead, including excessive ROS generation, mitochondrial dysfunction, autophagy activation, and inflammatory signaling [50,51,52,53]. However, Ni exhibits a distinct propensity to affect gastrointestinal tissues due to its frequent dietary exposure and close interaction with the gut immune microenvironment. These similarities suggest that the protective role of SelM in regulating redox balance, autophagy, and inflammation may not be unique to Ni stress, but could extend to other heavy metal toxicities. Further studies are warranted to explore whether SelM or other selenoproteins provide comparable protection against cadmium-, copper-, or lead-induced intestinal injury.

In summary, our study provides robust evidence that SelM plays a protective role in the colon against Ni-induced damage. By attenuating oxidative stress, restraining excessive autophagy, and modulating inflammatory signaling, SelM maintains colonic integrity under toxic insult. The regulatory effects of SelM align with the established roles of other selenoproteins and support its classification as a central redox and immune modulator. For example, glutathione peroxidases (GPX1 and GPX2) protect the intestinal epithelium from oxidative stress by reducing hydrogen peroxide and lipid peroxides [54], while thioredoxin reductase 1 (TXNRD1) is essential for maintaining intestinal redox homeostasis [55]. In addition, selenium-binding protein 1 (SBP1) has been implicated in regulating oxidative stress through its interaction with glutathione peroxidase 1, and reduced SBP1 expression has been associated with enhanced oxidative damage and disease progression [56]. These parallels suggest that SelM operates in concert with other selenoproteins to safeguard intestinal health, although its specific role in modulating the oxidative stress–autophagy–inflammation axis appears to be unique. Given the growing environmental and health concerns surrounding heavy metal exposure, our findings highlight the potential of targeting selenoprotein pathways for therapeutic interventions. From a practical perspective, selenium supplementation or approaches to enhance selenoprotein function may represent promising strategies to protect intestinal health and improve animal welfare under heavy metal stress, which is of particular relevance to livestock production and veterinary medicine. Future studies are warranted to dissect the precise molecular interactions involving SelM and its downstream effectors, which may yield novel strategies for preventing or treating metal-induced gastrointestinal diseases.

## 5. Conclusions

This study demonstrates that the colon is a primary target organ of nickel (Ni) toxicity. Ni exposure induces oxidative stress, which in turn activates autophagy in murine colonic tissues. Excessive autophagy further amplifies inflammatory responses, contributing to tissue damage. Notably, deletion of the SelM exacerbates these pathological alterations. Our findings indicate that SelM mitigates Ni-induced colonic inflammation by attenuating oxidative stress–driven autophagy. These results provide mechanistic insight into the interplay between redox imbalance, autophagy, and inflammation in heavy metal–induced intestinal injury, offering a foundation for future research in comparative pathology and clinical intervention. Furthermore, this study underscores the critical role of selenoproteins such as SelM in maintaining intestinal health under environmental stress and suggests that selenium-based nutritional strategies may enhance animal welfare and resilience in intensive farming systems challenged by heavy metal exposure.

## Figures and Tables

**Table 1:** The primers used in the present study.

Gene	Forward Primer (5′-3′)	Reverse Primer (5′-3′)
β-actin	CCAGCCATGTATGTAGCCATCCAG	AACACCATCACCAGAGTCCATCAC
ATG5	CAAGGATGCGGTTGAGGC	TGAGTTTCCGGTTGATGG
ATG7	TCGAAAACCCCATGCTCCTC	AGGGCCTGGATCTGTTTTGG
Beclin1	TGCAGGTGAGCTTCGTGTG	GCTCCTCTCCTGAGTTAGCCT
LC3	CTTCGCCGACCGCTGTAA	GCCGGATGATCTTGACCAACT
ATG1	GAAAGACAACGGACAAATCACC	GGGGGTGATATGTTTGAACTTG
mTOR	CAGACTGGCTCTTGCTCATAA	GCTGGAAGGCGTCAATC
PI3K	CTGGGGGACATACTGACTGT	GTTCCTGGAAAGTCTCCCCTC
NF-κB	ACTCTGTTTTGCACCTCGCT	TTCAGACCTTCACCGTTGGG
iNOS	ACTGGGTTGAATCTGGGTGA	GGAGTCTGGAGATTTCTTTGCTG
IL-18	CAAAGTGCCAGTGAACCCCAGAC	ACAGAGAGGGTCACAGCCAGTC
IL-1β	CTTCATCTTCTACCGCCTGGACAG	CTGGTCGGGTTGGTTGGTGATG
COX-2	CTGGCTCGGCACACTGATGATG	GCCCACGGACCAAATATCCACT
TNF-α	ACCACGCTCTTCTGCCTACT	GCTTTGACATTGGCTACAACG
IL-2	GCAGAATGGCTACGACACCG	CACTATGCTGGACAGGCAG
IL-6	CGGATGCTTCCAATCTGGGT	CAGGTGCCCCAGCTACATTA
IL-17	CCCAGATAGAAAGCGACTGC	ACCTCCTTGACGATGATGCT
IFN-γ	ATCCCATCCTCCGTTGTCCT	ATCGTCGCCTTCTTCGAGTT
IL-10	AGAGTGCCTTTAGCAAGCTCC	TAGAGTCGTCATCCTGGAAGGT

## Data Availability

The original contributions presented in this study are included in the article/Supplementary Material. Further inquiries can be directed to the corresponding author.

## Supplementary Materials

The following supporting information can be downloaded at: https://www.mdpi.com/article/10.3390/vetsci12100955/s1, Table S1: Summary of in vitro treatment groups (siRNA transfection, treatments, concentrations, and durations). File S1 Original images of western blot in this manuscript.
