# Supplementary material for: Selenoprotein M Protects Intestinal Health in Nickel-Exposed Mice: Implications for Animal Welfare Under Heavy Metal Stress
Source: Vet Sci. 2025 Oct 4;12(10):955. doi: 10.3390/vetsci12100955 (PMC12567978; doi:10.3390/vetsci12100955)
Supplement: Supplementary file 1 [file vetsci-12-00955-s001.zip › File S1 Original images of western blot.pdf]

Location	Protein Name	Molecular Weight	Origin Image
Figure 3B	LC3-II	16 kDa	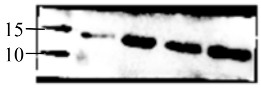
Figure 3B	ATG-7	77 kDa	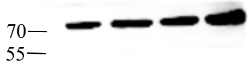
Figure 3B	P62	62 kDa	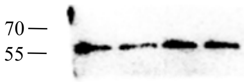
Figure 3B	Beclin1	55 kDa	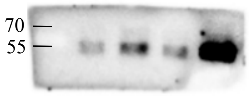
Figure 3B	β-actin	42 kDa	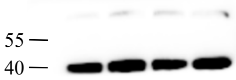
Figure 3G	Beclin1	55 kDa	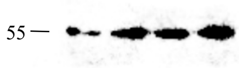
Figure 3G	ATG-7	77 kDa	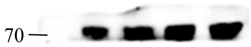
Figure 3G	LC3-II	16 kDa	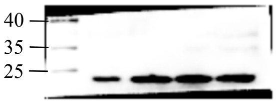
Figure 3G	β-actin	42 kDa	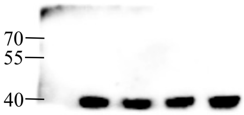
Figure 4B	TNF-α	17 kDa	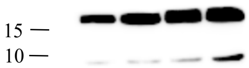
Figure 4B	IL-10	18 kDa	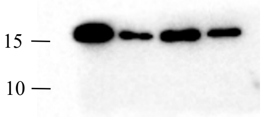
Figure 4B	IL-1β	17 kDa	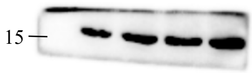
Figure 4B	NF-κB	115 kDa	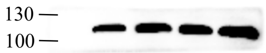

Figure 4B	β-actin	42 kDa	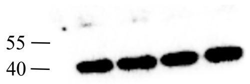
Figure 4E	TNF-α	17 kDa	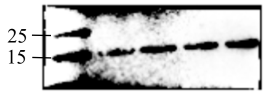
Figure 4E	IL-10	18 kDa	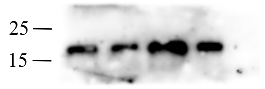
Figure 4E	IL-1β	17 kDa	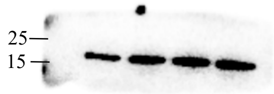
Figure 4E	IL-2	15 kDa	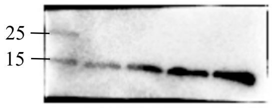
Figure 4E	β-actin	42 kDa	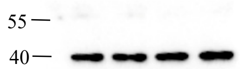
Figure 5F	ATG-7	77 kDa	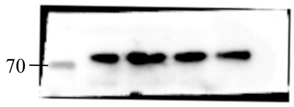
Figure 5F	LC3-II	16 kDa	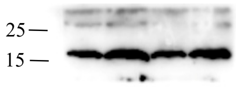
Figure 5F	IL-1β	17 kDa	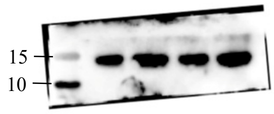
Figure 5F	TNF-α	17 kDa	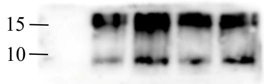
Figure 5F	β-actin	42 kDa	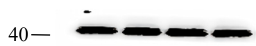
