# Supplementary material for: Selenoprotein M Protects Intestinal Health in Nickel-Exposed Mice: Implications for Animal Welfare Under Heavy Metal Stress
Source: Vet Sci. 2025 Oct 4;12(10):955. doi: 10.3390/vetsci12100955 (PMC12567978; doi:10.3390/vetsci12100955)
Supplement: Supplementary file 1 [file vetsci-12-00955-s001.zip › Supplementary Table S1.docx.pdf]

Table S1: Summary of in vitro treatment groups (siRNA transfection, treatments, concentrations, and

Group	siRNA transfection	Treatment	Concentration	Duration
Control	si-NC	None	–	–
Ni	si-NC	$NiCl_2$	5 μM	12 h
si-SelM	si-SelM	None	–	–
si-SelM + Ni	si-SelM	$NiCl_2$	5 μM	12 h
si-SelM + Ni + NAC	si-SelM	NAC (pre-treatment) + $NiCl_2$	NAC: 2.5 mM (2 h) → $NiCl_2$: 5 μM (12 h)	2h pre-treatment + 12 h $NiCl_2$
si-SelM + Ni + 3-MA	si-SelM	3-MA (pre-treatment) + $NiCl_2$	3-MA: 5 mM (2 h) → $NiCl_2$: 5 μM (12 h)	2h pre-treatment + 12 h $NiCl_2$

durations).
